# Structure based release kinetics analysis of doxazosin mesylate sustained-release tablets using micro-computed tomography

**DOI:** 10.1016/j.ajps.2024.100966

**Published:** 2024-09-21

**Authors:** Qian Liu, Mengqing Zan, Hanhan Huang, Hai Su, Wenjing Zhang, Lingyun Ma, Guangchao Zhang, Zunjian Zhang, Jiwen Zhang, Jianzhao Niu, Mingdi Xu

**Affiliations:** a NMPA Key Laboratory for Quality Research and Evaluation of Chemical Drugs, National Institutes for Food and Drug Control, Beijing 100050, China; b China Pharmaceutical University, Nanjing 210009, China; c NMPA Key Laboratory for Quality Research and Evaluation of Pharmaceutical Excipients, National Institutes for Food and Drug Control, Beijing 100050, China; d Center for Drug Delivery System, Shanghai Institute of Materia Medica, Chinese Academy of Sciences, Shanghai 201203, China

**Keywords:** Doxazosin mesylate sustained-release tablets, Osmotic pump tablets, Micro-computed tomography, Three-dimensional structures, Ethanol

## Abstract

The structures of solid dosage forms determine their release behaviors and are critical attributes for the design and evaluation of the solid dosage forms. Here, the 3D structures of doxazosin mesylate sustained-release tablets were parallelly assessed by micro-computed tomography (micro-CT). There were no significant differences observed in the release profiles between the RLD and the generic formulation in the conventional dissolution, but the generic preparation released slightly faster in media with ethanol during an alcohol-induced dose-dumping test. With their 3D structures obtained via micro-CT determination, the unique release behaviors of both RLD and the generic were investigated to reveal the effects of internal fine structure on the release kinetics. The structural parameters for both preparations were similar in conventional dissolution test, while the dissolutions in ethanol media showed some distinctions between RLD and generic preparations due to their static and dynamic structures. Furthermore, the findings revealed that the presence of ethanol accelerated dissolution and induced changes in internal structure of both RLD and generic preparations. Moreover, structure parameters like volume and area of outer contour, remaining solid volume and cavity volume were not equivalent between the two formulations in 40 % ethanol. In conclusion, the structure data obtained from this study provided valuable insights into the diverse release behaviors observed in various modified-release formulations in drug development and quality control.

## Graphical abstract

**Figure:**
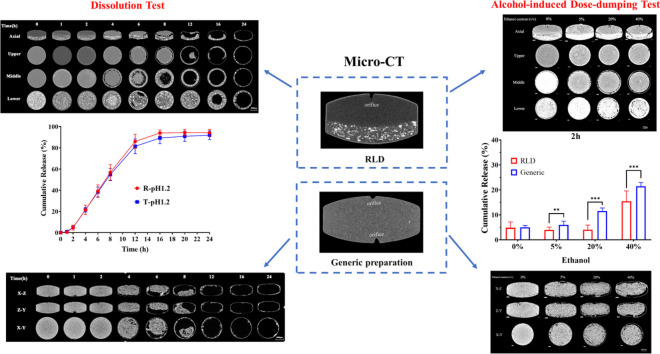

## 1 Introduction

The similarity evaluation is highly important for both the industry and academy, since more than 97 % of launched chemical drugs are generics, with only a small portion being modified release formulations [[1], [2], [3]]. The osmotic pump system has been a popular and reliable technique with unique advances, including independence from gastrointestinal pH variations and non-disturbance by concomitant food intake during drug release, ensuring the drug concentration in plasma within the therapeutic range over an extended duration. As a result, the dosing frequency can be reduced, leading to improved patient compliance. However, it was limited to making generics due to its complicity in the production process, quality control and other specific know-hows [[4], [5], [6]]. Even if the reference listed drug (RLD) and the generic osmotic pump system exhibit a similarity *in vitro* release kinetics, the bioequivalence remains uncertain. Therefore, the design of the preparation and thorough evaluation of critical quality attributes are crucial factors.

The structure of the osmotic pump systems, along with the sub-unit compositions and granular structure essential for tablet formation, represents the intrinsic characteristics of advanced solid dosage forms. These elements significantly impact the quality and effectiveness of final pharmaceutical products. While the structure plays a crucial role in osmotic systems, current research, development, production processes, and quality control have not yet fully mastered the internal structure of osmotic pump systems. [7,8]. Other than macro structure observations with naked eyes or optical photos, noninvasive imaging is the most intuitive way to directly observe the internal structure of solid dosage forms. High-resolution imaging tools like micro-computed tomography (micro-CT) visualize the internal architecture in three-dimensional (3D) and the dynamic structural evolutions the osmotic pump systems undergo during drug release phase [[9], [10], [11], [12]]. In combination with structure 3D reconstruction, micro-CT is capable of visualizing the static and dynamic 3D structure of the preparation in high resolution [[13], [14], [15]], which has been applied in the characterization of the internal 3D structure of solid preparations [16,17].

Doxazosin mesylate, a type of α1 adrenergic receptor blocker, is a biopharmaceutics classification system (BCS) class I drug used in the clinical treatment of benign prostatic hyperplasia and hypertension. The RLD of doxazosin mesylate sustained release tablets (DM SRT) is Cardura XL®, a brand product of Pfizer Inc., with a typical bilayer structure of osmotic pump system, whilst there is only one generic product available on the market in China, namely, Liheng®, produced by Hefei Lifeon Pharmaceutical Co., Ltd., China. Abi Maharjan [12] reported insight 3D static and dynamic structure investigation of the RLD of DM SRT using synchrotron radiation micro-computed tomography (SR-µCT) in Shanghai Synchrotron Radiation Facility (SSRF), which is an advanced imaging method with less accessibility for the industry compared to desktop micro-CT. In this study, the release characteristics of RLD and the generic product of DM SRT were investigated in combination of dissolution test and micro-CT structure characterization. The release mechanisms of DM SRT were elucidated based on their 3D structure and the correlation of the cumulative releases and the 3D structural parameters was established. Moreover, the effects of ethanol on the microstructural changes of osmotic pump tablets will be visualized, providing insights for the development and quality control of oral controlled release generic drugs.

## 2 Materials and methods

### 2.1 Materials

Cardura XL (4 mg doxazosin per tablet) was purchased from Pfizer Inc. (Batch number ET6766), in which doxazosin mesylate is the active pharmaceutical ingredient (API), with the excipients including polyoxyethylene (PEO), sodium chloride (NaCl), hydroxypropyl methylcellulose (HPMC), iron red oxide, magnesium stearate, cellulose acetate, polyethylene glycol, white and black ink. Liheng® was purchased from Hefei Lifeon Pharmaceutical Co., Ltd, China (Batch number 210507), with one orifice on both the upper and lower side of the coated membrane to promote drug release, in which the excipients are NaCl, HPMC, cellulose acetate, magnesium stearate, etc. Micro-CT (SKYSCAN 2214) was provided by Bruker (Karlsruhe, Germany). All solvents were of chromatography grade. All other chemicals and reagents were analytical grade.

### 2.2 *In vitro* dissolution test

The *in vitro* release profiles of 12 tablets each of RLD and generic DM SRT were tested in three dissolution media (0.1 M hydrochloric acid, pH 1.2; acetate buffer, pH 4.5 and phosphate buffer, pH 6.8), sampled after 1, 2, 4, 6, 8, 12, 16, 20 and 24 h. The similarity factor (*f_2_*) can be used as an evaluation parameter of dissolution difference between the RLD and the generic preparation.

### 2.3 Dose dumping induced by ethanol *in vitro*

According to Product-Specific Guidance for Generic Drug Development published by Food and Drug Administration (FDA, USA) and National Medical Products Administration (NPMA, China) [18,19], the drug release behaviors were investigated to 12 dosage units of DM SRT in a media of hydrochloric acid solution containing 5 %, 20 % and 40 % ethanol or without ethanol at the conditions of *in vitro* dissolution test. Details of the method are provided in the supplementary materials.

### 2.4 Preparation of samples and acquisition of tablet projections by micro-CT

Tablet samples for static structure analysis were randomly selected directly from RLD and generic DM SRT. Then, the tablets for dynamic structure analysis were sampled from the dissolution media at predetermined time points according to the conditions of *in vitro* dissolution test. In order to eliminate the influence of dissolution media on imaging, filter paper was used to absorb the residual dissolution media on the sample surface immediately. Then, it was dried in an airtight vessel containing dried allochronic silica gel at room temperature for 72 h before scanning.

### 2.5 3D structural reconstruction and quantification of DM SRT

Micro-CT can be used for micro-imaging of middle and low-density samples with high resolution. According to the size of DM SRT and the analysis target, the effective pixel size of the Cardura® XL was set as 6.0 µm (current: 150 µA, voltage: 70 kV, exposure time: 0.4 s), and the effective pixel size of Liheng® was also set as 6.0 µm (current: 150 µA, voltage: 70 kV, exposure time: 0.32 s). For each measurement, the sample was fixed on the stage, rotated by 0.2° for each projection and scanned by 180°.A total of 1800 images were obtained. Data processing includes two parts. First, the acquired images were imported into NRecon software (version 2.1.0, Bruker, Belgium) for 3D reconstruction. Meanwhile, CTAn software (version 1.20.8, Bruker, Belgium) was used to extract region of interest (ROI) and related 3D structural parameters were calculated.

### 2.6 Statistical analysis

Statistical analyses were performed utilizing GraphPad Prism 8.0.1 (La Jolla, CA, USA) and the significance levels were evaluated using a two-way *t*-test. Coefficient of determination (*R^2^*) was calculated by Excel (Microsoft 365).

## 3 Results and discussion

### 3.1 *In vitro* release kinetics of RLD and the generic DM SRT

**Fig. 1:**
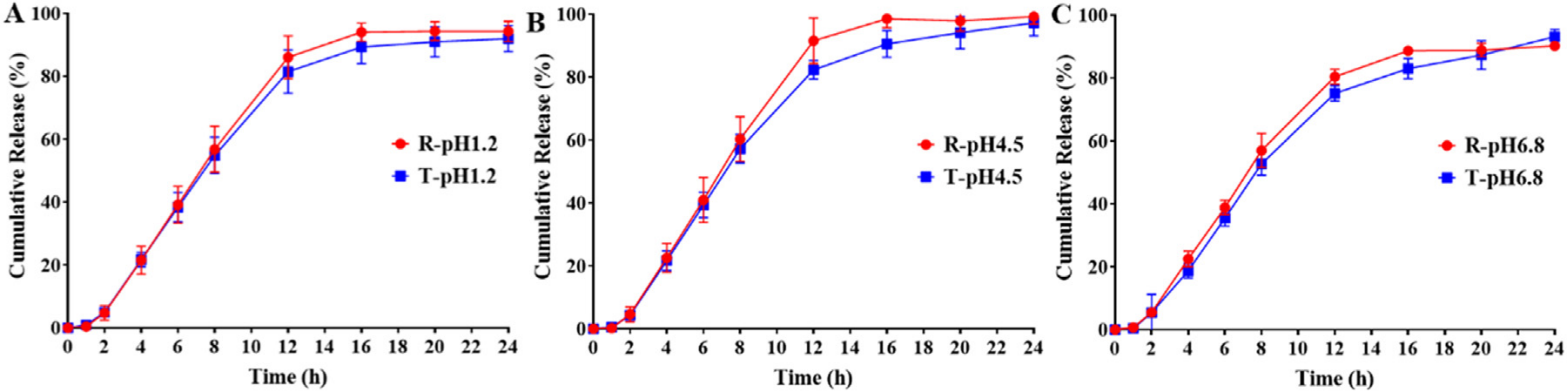
Release profiles of RLD and generic DM SRT in different dissolution media: (A) pH 1.2, (B) pH 4.5, and (C) pH 6.8.

To compare the release similarity of RLD and generic of DM SRT, conventional dissolution tests *in vitro* following FDA-recommended dissolution methods were conducted first. As shown in Fig. 1, more than 90 % dose of the RLD and generic DM SRT were released within 24 h in three different dissolution media. The dissolution profile of generic preparations exhibited high similarity to the RLD, with the *f_2_* of 77.0, 57.1 and 71.6 in three media with pH of 1.2, 4.5 and 6.8, respectively. The release similarity of RLD and the generic DM SRT *in vitro* was consistent with the bioequivalence *in vivo* published by Li et al. [20]. At the early stage within 2 h, only about 5 % dose of RLD and the generic were released. Then, the release rate of both RLD and generic DM SRT increased dramatically from 4 h and reached the peak at 12 h. The cumulative release percentages of RLD at 24 h reached 94.3 %, 99.2 % and 90.2 % in three media with pH of 1.2, 4.5 and 6.8, respectively, while the cumulative release percentages of the generic at 24 h reached 91.3 %, 97.2 % and 93.1 %, respectively. These data exhibited the basic release properties of sustained release tablets in conventional dissolution test.

**Fig. 2:**
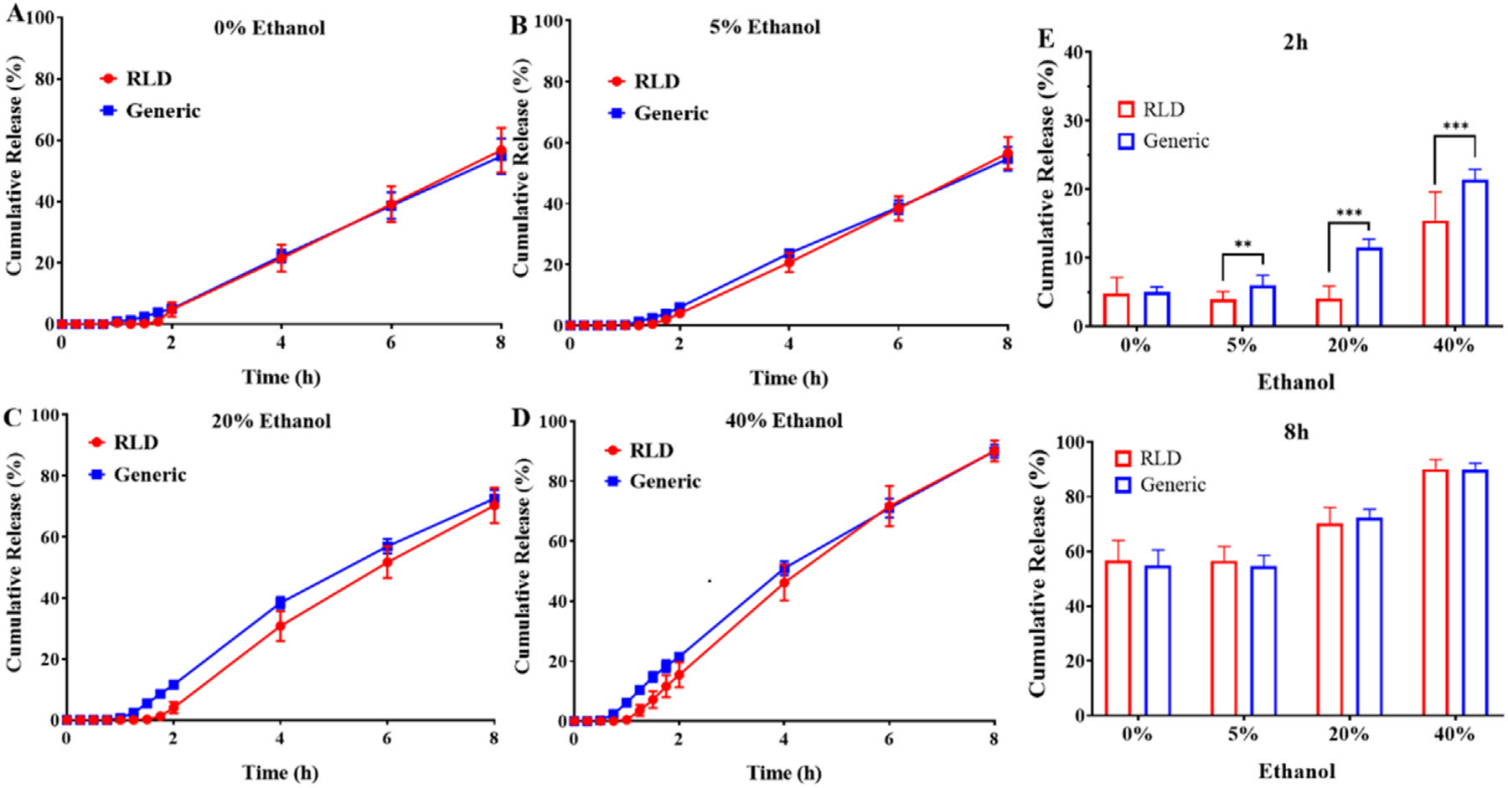
Release profiles of RLD and generic DM SRT in dissolution media with or without ethanol. (A) Conventional dissolution media (pH 1.2). (B) dissolution media (pH 1.2) containing 5 % ethanol. (C) Dissolution media (pH 1.2) containing 20 % ethanol. (D) Dissolution media (pH 1.2) containing 40 % ethanol. (E) cumulative release percentage of DM in various content of ethanol dissolution at 2 h and 8 h; *t*-test, ^⁎⁎^*P* < 0.01, ^⁎⁎⁎^*P* < 0.001.

Moreover, due to a concern about dose dumping when sustained release tablets are taken with alcohol, regulatory authorities, including FDA, the European Medicines Agency (EMA) and NMPA, issued guidelines to suggest additional dissolution test using various concentrations of ethanol in the dissolution media [18,19]. Thus, the release behavior of both the RLD and generic of DM SRT were further evaluated in dissolution media pH 1.2 with ethanol. The release of doxazosin mesylate in RLD and the generic groups increased following ethanol concentrations in the dissolution media rose from 5 % to 40 % at 8 h. However, the cumulative release of doxazosin mesylate in the generic group significantly surpassed compared to RLD group at the same concentrations of ethanol at 2 h, with 5.98 % *vs.* 3.97 % (5 % ethanol), 11.57 % *vs.* 4.04 % (20 % ethanol) and 21.46 % *vs.* 15.43 % (40 % ethanol), respectively (Fig. 2), in spite of the value of *f_2_* factor greater than 50. When the experiment time was extended to 8 h, the cumulative release percentages of doxazosin mesylate of RLD and the generic group in dissolution media pH 1.2 with 40 % ethanol were almost the same with 90.06 % and 90.02 %, respectively, and the value of *f_2_* factor is 65.7 (Fig. 2). Taken together, it is necessary to investigate the underlying reasons to these observations.

### 3.2 Static structures of RLD and generic preparations

**Fig. 3:**
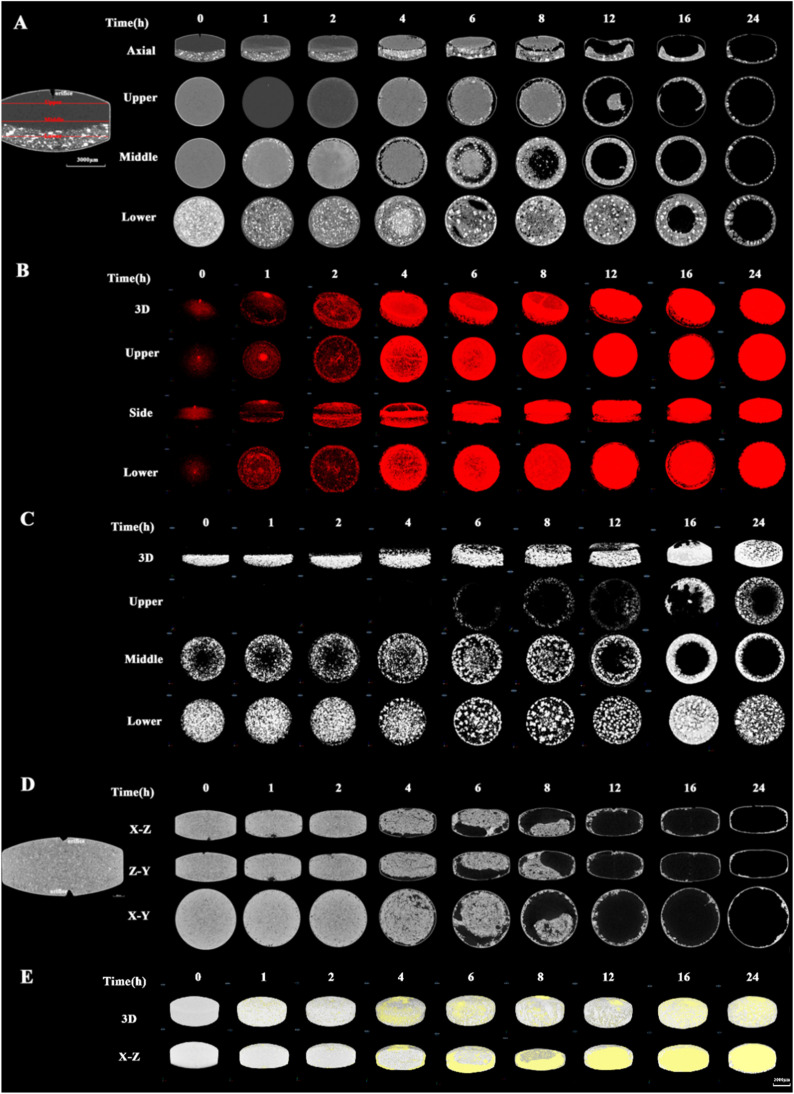
Internal microstructure of the RLD and generic DM SRT during dissolution. 2D section diagram of the (A) RLD and (D) generic DM SRT; upper, middle and lower represent the radial slice at the respective position. Spatial distribution of (B) voids and (C) sodium chloride in the RLD (sodium chloride in white). (E) Spatial distribution of voids and solid contents in the generic DM SRT (gray is solid content, yellow is voids). (For interpretation of the references to color in this figure legend, the reader is referred to the web version of this article.)

The structure of sustained release tablets represented their intrinsic characteristics. By micro-CT test to measure the structure of DM SRT, it is found that RLD and the generic had diverse static structures (Fig. 3A and 3D). The RLD tablet presented as a bilayer osmotic pump of “drug layer-push layer” with a drilled orifice outside of the drug layer, while the generic tablet was a “monolith osmotic pump tablet” with one drilled orifice to each of the upper side and lower side of the tablets. The process of bilayer osmotic pump system of RLD tablet is to mix the drug-containing layer and the push layer respectively for granulation. The drug-containing layer was pre-pressed, and then the push layer materials were added for secondary pressing. Notably, there is a high-density region of NaCl (salt crystal) in pushing layer (lower) of RLD preparation (Fig. 3A). The process of monolayer osmotic pump system of generic tablet is to mix the API and excipients for granulation, and the tablet is pressed only once. The generic tablets appear to have an even distribution of density inside (Fig. 3D).

### 3.3 Dynamic structures of RLD and generic preparations in conventional dissolution

Both RLD and generic preparations of DM SRT were measured for the dynamic structure changes in conventional dissolution test, respectively. As shown in Fig. 3A, the internal structure of RLD tablets showed typical transformation in a sectioned axial view at 6 h. A hollow space appeared between the drug layer and the pushing layer, and it finally disappeared at 12 h. A “U” shaped morphological structure in the pushing layer was also observed at 6 h and enlarged after. This “U” shaped structure was formed after the push layer had absorbed water and expanded. Spatial distribution of voids also showed that the drug moved up and released from the peripheral area of the tablet through the delivery orifice (Fig. 3B). Salt crystals were similarly found in the push layer of the tablet, where they established an osmotic gradient facilitating water penetration. Spatial distribution of the crystals revealed a gradual movement of the drug layer from the periphery towards release through the orifice, ultimately resulting in the U-shaped push layer remaining (Fig. 3C). The crystals exhibited varying effects in different sections of the push layer. The salt near the peripheral region of the tablet was initially dissolved by water, forming a salt solution of specific concentration. The disparity in osmotic pressure between the interior and exterior of the semipermeable membrane creates a driving force that promotes the influx of water into the membrane. The high molecular weight PEO in the push layer absorbed water and expanded to push the drug out from the orifice. The salt in the center area of the push layer contacted with less water and formed a higher concentration of salt solution. Due to the concentration gradient between the salt and the peripheral area, there was a phenomenon of the diffusion of high concentration of salt to the area with low concentration of salt. The high mobility made the peripheral region move towards the drug layer and eventually promoted an increased drug release velocity of the drug layer. However, the drug release velocity decreased at the later stage due to the lower drug content and the maximum swelling capacity of high molecular weight PEO and low molecular weight PEO.

The dynamic structures and images of the generic DM SRT as a monolithic osmotic pump in dissolution test were simultaneously monitored by micro-CT. At the initial stage, minimal change was observed in both orifices, indicating that the semipermeable membrane is rigid and could withstand the free access of water effectively. However, some little cracks could be seen in the tablet core and areas close to the orifices (1 h and 2 h). The shapes of the orifices gradually changed from the initial funnel shape to the irregular shape and finally disappeared at 4 h, which were attributed to the dissolution to the solid core (Fig. 3D). During the drug release process, the solid contents close to the orifices disappeared more quickly than elsewhere, indicating that the drug and excipients were dissolved or released more quickly there. Most of the solid contents had vanished at 12 h, and only a small quantity of residual solids was attached to the semipermeable membrane. Meanwhile, voids close to orifices accumulated at 4 h and distributed nearly whole space of the table core at 24 h (Fig. 3E).

### 3.4 Quantitative analysis of RLD and generic DM SRT in structural parameters

**Fig. 4:**
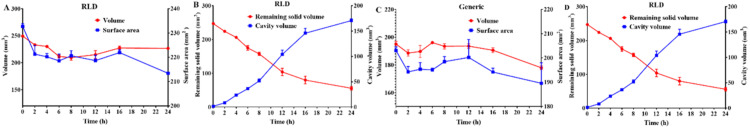
3D structural parameter profiles of RLD and generic DM SRT over time. (A) Outer contour volume and surface area of the RLD. (B) Remained solid volume and cavity volume of RLD. (C) Outer contour volume and surface area of the generic preparation. (D) Remained solid volume and cavity volume of the generic preparation. (*n* = 3).

**Fig. 5:**
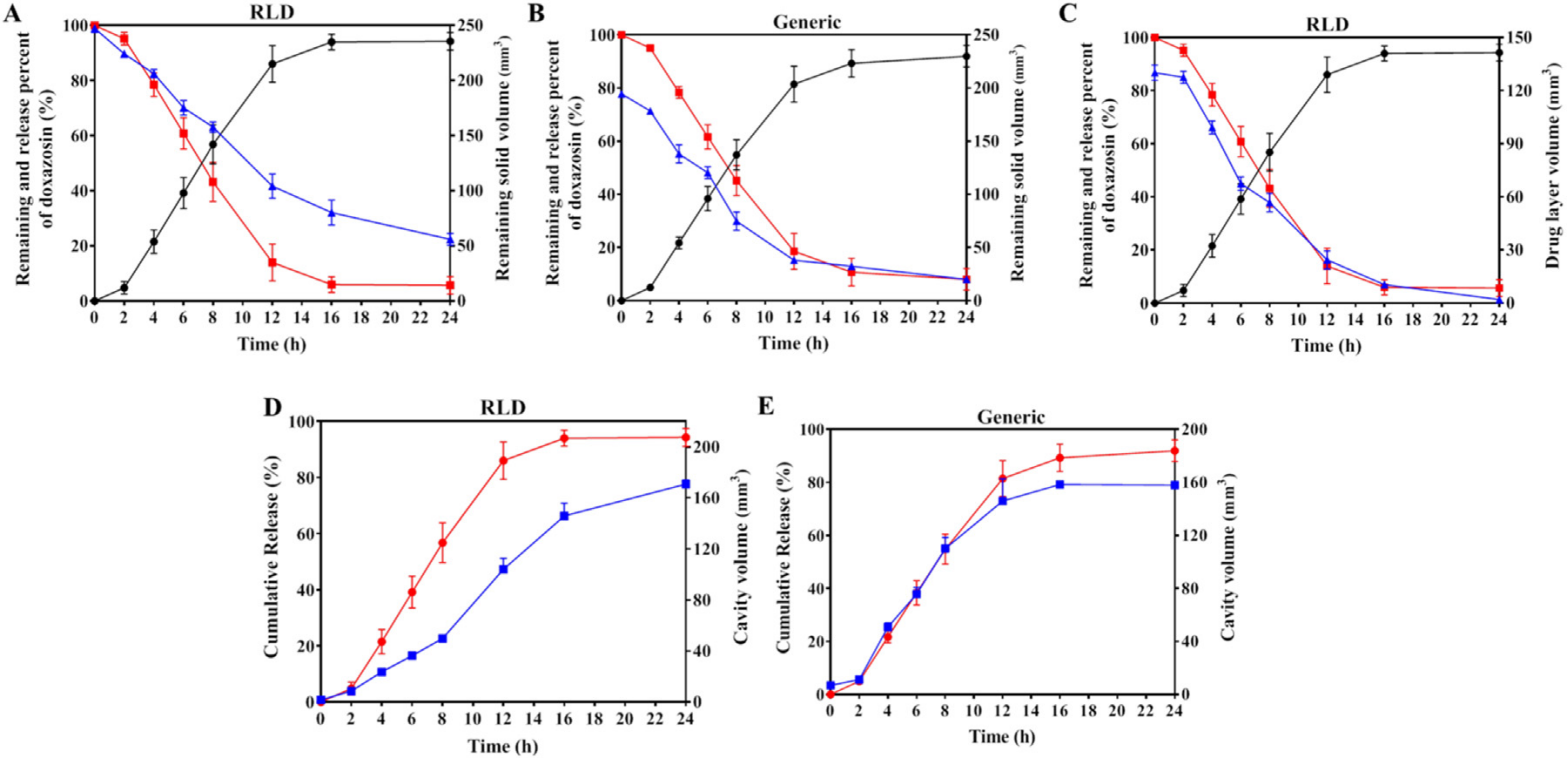
Correlation of released percentages and remaining percentages of doxazosin with 3D structural parameters of the DM SRT. (A and B) released percentages and remaining percentages of doxazosin in the RLD and generic DM SRT preparation plotted with remaining solid volume (black is the release percent of doxazosin, red is the remaining percent of doxazosin, blue is the remaining solid volume). (C) Released percentages and remaining percentages of doxazosin in the RLD plotted with drug layer volume (black is the release percent of doxazosin, red is the remaining percent of doxazosin, blue is drug layer volume). (D and E) Release percentages of doxazosin in the RLD and generic DM SRT plotted with cavity volume (red is the release percent of doxazosin, and blue is the cavity volume). (For interpretation of the references to color in this figure legend, the reader is referred to the web version of this article.)

Although the dynamic structure behaviors of RLD distinguished from that of generic DM SRT in structures, there were no significant differences of structural parameters as surface area and cavity volume for both preparations (Fig. 4A–4D). The dynamic tendencies of parameters like volume and area of outer contour, remaining solid volume and cavity volume were similar for both RLD and generic preparations. The remaining drug contents in both RLD and the generic tablets were well linear correlated with the remaining solid volume (*R^2^* = 0.972 and 0.989, respectively) (Fig. 5A and 5B). The cumulative release percentages of both RLD and the generic preparations were linearly correlated with the cavity volume (*R^2^* = 0.900 and 0.993, respectively) (Fig. 5D and 5E). Thus, these data supported the similarities of RLD and generic DM SRT for their *in vitro* release profile as well as their dynamic structures.

### 3.5 Dynamic structures of RLD and generic preparations in alcohol-induced dose-dumping test

**Fig. 6:**
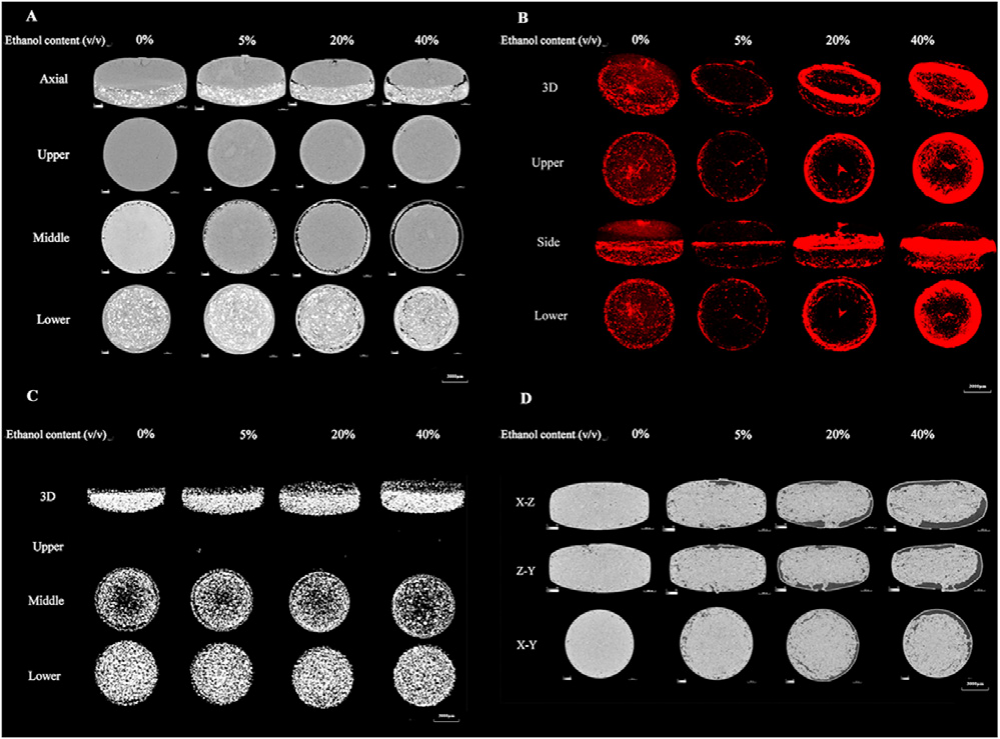
Internal microstructure of the DM SRT in dissolution media (pH 1.2) containing ethanol for 2 h. (A) Two-dimensional section diagram of the RLD at different ethanol concentrations. (B) Voids distribution of the RLD at different ethanol concentrations (voids in red). (C) Spatial distribution of sodium chloride in the RLD at different ethanol concentrations (white is sodium chloride). (D) Three views of generic DM SRT at different ethanol concentrations. (For interpretation of the references to color in this figure legend, the reader is referred to the web version of this article.)

The release profiles of the RLD and the generic DM SRT preparation were generally similar in alcohol-induced dose-dumping test in view of the value of *f_2_* more than 50, indicating that the release profiles alone could not distinguish the alcohol tolerance degree of the two types of osmotic pump systems in internal structure. Therefore, the dynamic structures of RLD and generic DM SRT preparations in various alcohol solutions were evaluated. The RLD tablets were tested in the dissolution media containing varying concentrations of ethanol within 2 h and subsequently dried for 72 h to acquire 2D images. Both the drug layer and push layer of the RLD changed to an extent as the ethanol concentration increased, accompanied by an increase in the volume of cracks in the push layerʼs peripheral region (Fig. 6A and 6C). Ethanol possibly increased drug release by affecting the expansion rate of the high molecular material near the orifice, but it has no effect on the drug release pathway. Furthermore, higher ethanol concentrations resulted in the generation of larger voids in the drug layer near the periphery of the push layer (Fig. 6B), correlating with an increase in cumulative release. Similarly, the performance of the generic preparation was assessed using micro-CT in alcohol-induced dose-dumping test simultaneously. The voids volume of the monolayer osmotic pump tablet changed to some extent as the ethanol concentration increased, with voids near the delivery orifices expanding with increasing ethanol concentration (Fig. 6D).

Also, the statistical analysis was performed for the 3D structural parameters of the samples (*n* = 3) of both RLD and the generic DM SRT without ethanol and with 5 %, 20 % and 40 % ethanol. The data of RLD showed that there were significant differences in the remaining solid volume, cavity volume, drug-layer volume and The surface area of push layer after adding ethanol (*P* < 0.01) (Table 1). There were no significant differences between the two groups (without and with ethanol) in terms of volume, surface area, the surface area of drug layer or push layer volume. For the generic DM SRT, there were significant differences in the volume of the external contour, the surface area, the volume of the remaining solid content, the cavity volume, and the diameter after adding ethanol (*P* < 0.01) while no significant differences in the tablet thickness and semipermeable membrane thickness (Table 2).

**Table 1:** Comparison of 3D structure parameters of RLD in dissolution media with various concentrations of ethanol (*n* = 3).

Parameters	Ethanol			
	0	5 %	20 %	40 %
Volume (mm^3^)	233.1 ± 2.5	228.8 ± 0.4	233.8 ± 3.5	228.2 ± 4.2
Surface area (mm^2^)	221.3 ± 6.2	220.2 ± 1.0	229.1 ± 6.7	222.6 ± 0.6
Remaining solid volume (mm^3^)	224.3 ± 2.6	217.3 ± 2.6	217.3 ± 5.9	203.2 ± 3.8^⁎⁎^
Cavity volume (mm^3^)	8.5 ± 0.3	11.5 ± 2.4	16.5 ± 2.6^⁎⁎^	24.7 ± 1.4^⁎⁎⁎^
Drug layer volume (mm^3^)	127.6 ± 3.3	112.6 ± 2.1^⁎⁎^	110.4 ± 5.6	105.9 ± 3.3^⁎⁎⁎^
The surface area of drug layer (mm^2^)	1401.2 ± 71.7	1332.3 ± 19.7	1314.1 ± 13.0	1514.7 ± 34.6
Push layer volume (mm^3^)	97.1 ± 3.8	89.2 ± 1.2	91.7 ± 0.7	97.3 ± 0.5
The surface area of push layer (mm^2^)	511.5 ± 21.0	569.1 ± 77.2	761.5 ± 74.2^⁎⁎^	890.5 ± 61.4^⁎⁎⁎^

**P < 0.01, *** *P* < 0.001 *vs.* 0 ethanol groups of reference preparations.

**Table 2:** Comparison of 3D structure parameters of generic preparations in dissolution media without ethanol and various concentrations of ethanol (*n* = 3).

Parameters	Ethanol			
	0	5 %	20 %	40 %
Volume (mm^3^)	188.1 ± 0.5	190.0 ± 2.9	194.1 ± 2.6	199.1 ± 1.3^⁎⁎^
Surface area (mm^2^)	194.1 ± 1.3	194.2 ± 1.8	197.4 ± 3.8	205.1 ± 1.7^⁎⁎^
Remaining solid volume (mm^3^)	172.1 ± 5.4	173.7 ± 4.2	158.9 ± 3.9	133.1 ± 2.5^⁎⁎⁎^
Cavity volume (mm^3^)	15.9 ± 4.9	16.2 ± 1.3	35.3 ± 1.5^⁎⁎^	66.0 ± 8.0^⁎⁎⁎^
Diameter (mm)	8.59 ± 0.02	8.57 ± 0.03	8.72 ± 0.02^⁎⁎^	8.75 ± 0.51^⁎⁎^
Tablet thickness (mm)	4.26 ± 0.10	4.29 ± 0.08	4.32 ± 0.09	4.35 ± 1.82
Semipermeable membrane thickness (mm)	0.060 ± 0.004	0.061 ± 0.002	0.065 ± 0.003	0.071 ± 5.078

** *P* < 0.01, *** *P* < 0.001 *vs.* 0 ethanol groups of generic preparations.

The statistical analysis was further performed for the 3D structural parameters of the RLD and generic DM SRT preparation in medium containing 40 % ethanol using the two-sample *t*-test (Table 3), respectively. The data showed that there were significant differences in the volume and area of outer contour, remaining solid volume and cavity volume between two groups of samples (*P* < 0.01). These structural features led to the different release phenomena of both RLD and generic DM SRT preparations in ethanol-containing media.

**Table 3:** Comparison of 3D structure parameters of the RLD and generic DM SRT in media containing 40 % ethanol (*n* = 3).

Parameters	RLD	Generic	*P*-value
Volume (mm^3^)	228.2 ± 4.2	199.1 ± 1.3	0.0005
Surface area (mm^2^)	222.6 ± 0.6	205.1 ± 1.7	0.0011
Remaining solid volume (mm^3^)	203.2 ± 3.8	133.1 ± 2.5	0.0000
Cavity volume (mm^3^)	24.7 ± 1.4	66.0 ± 8.0	0.0002

Both the RLD and the generic preparation of DM SRT can resist the dose dumping induced by alcohol in a certain range. According to Jedinger et al. [21], the mechanism of resistance to ethanol-induced dose dumping may be as follows. Firstly, both the bilayer and the monolayer osmotically-controlled systems are designed to maintain controlled-release behaviors and protect the delivery system unaffected by ethanol. Secondly, the formulation of RLD contains PEO, a non-ionic homopolymer of ethylene oxide, which is soluble in water due to hydration of the ether oxygen and insoluble in alcohol. When PEO is exposed to water, it hydrates rapidly, expands significantly, and subsequently forms a hydrogel layer on the surface of the matrix. The *in vitro* drug release studies indicated that the PEO matrices remained intact, and no dose dumping effect was observed after exposure to 5 % and 40 % hydro-alcoholic media for up to 8 h. What's more, the formulations of RLD and generic DM SRT preparations both contain HPMC, a non-ionic water-soluble polymer with a pH-independent drug release profile. HPMC is soluble in cold water, but almost insoluble in ethanol and hot water, and thus unaffected by ethanol when co-existing with ethanol.

With the increase of alcohol concentration, the volume, surface area and semipermeable membrane thickness of the generic drug increased significantly, while these parameters of RLD did not change, which may be related to the process of coating, or may be related to the content and quality of cellulose acetate in the excipients. It was also reported that ethanol increased the permeability, elasticity and swelling of cellulose acetate membrane [21]. Taken together, Compared with RLD DM SDT, generic tablets have a stronger risk of ethanol-induced dose dumping.

## 4 Conclusion

In this study, the *in vitro* release characteristics of reference and generic DM SRT preparations were investigated by *in vitro* dissolution test. The micro-CT analysis evaluated the 3D static and dynamic structures of both at micron-scale resolution. It was found that the DM SRT generic and RLD had similar dynamic structure and consistent release kinetics characteristics, while the static structure of generic is a bit different from the RLD. The *f_2_* of the RLD and generic DM SRT preparations decreased from 80 to 63 in the conventional acid dissolution media and the acidic media with ethanol added, indicating the continued similarity in spite of the presence of alcohol and both types of the osmotic pump preparations could resist ethanol erosion to a certain extent. In this study, micro-CT was used to explore the structural mechanism of the ethanol influence on the osmotic pump tablets, and it was found that ethanol did not change the characteristics and internal passages of drug release but changed some 3D structural parameters of tablets, thus speeding up the drug release speed. Overall, structure-based release kinetics analysis using Micro-CT would be a useful tool for quality control and evaluation of osmotic pump controlled-release tablets.

Micro-CT could be served as a facile evaluation method for the 3D cross-scale visualization and structural quantification of the high-end osmotic pump systems with complex structures. The accelerated impact of alcohol presence on the release performance of both formulations can be visualized and determined intuitively. The structural consistency of generic products and RLD are well correlated with the release behaviors, which is a helpful support for the bioequivalence evaluation and quality control.

## Conflicts of interest

The authors declare no conflict of interest.

## Appendix Supplementary materials

Image, application 1
