# Supplementary material for: Structure based release kinetics analysis of doxazosin mesylate sustained-release tablets using micro-computed tomography
Source: Asian J Pharm Sci. 2024 Sep 21;19(6):100966. doi: 10.1016/j.ajps.2024.100966 (PMC11617939; doi:10.1016/j.ajps.2024.100966)
Supplement: Supplementary file 1 [file mmc1.docx]

**Supplementary material**

**Structure based Release Kinetics Analysis of Doxazosin Mesylate Sustained-Release Tablets Using Micro-Computed Tomography**

Qian Liu^a^, Mengqing Zan^a, b^, Hanhan Huang^a, b^, Hai Su^a, b^, Wenjing Zhang^a, b^, Lingyun Ma^a^, Guangchao Zhang^a^, Zunjian Zhang^b^, Jiwen Zhang^c,d*^, Jianzhao Niu^a*^ , Mingdi Xu^a*^

*In vitro dissolution test Method:*

The release profiles of 12 tablets each of RLD and generic DM SRT were tested using paddle method at 75 rpm in three dissolution media (0.1 M hydrochloric acid, pH 1.2; acetate buffer, pH 4.5 and phosphate buffer, pH 6.8). Aliquots of 5 ml were sampled after 1, 2, 4, 6, 8, 12, 16, 20 and 24 h, and the same volume of fresh dissolution media preheated to 37 ℃ ± 0.5 ℃ was added in time to maintain sink conditions, and 0.45 μm microporous filtration membrane was used for timely filtration to the samples. The samples were quantitatively analyzed by high performance liquid chromatography (column information: Agela Technologies Venusil MP C18, 4.6 mm ×150 mm, 5 μm), and the release rates were calculated thereafter. Chromatographic conditions were as follows: mobile phase consisted of acetonitrile (400:300) in aqueous solution containing 0.5% acetic acid (v/v) and 0.25% triethylamine (v/v), flow rate 0.6 ml/min, column temperature 30 ℃, detection wavelength 246 nm, an injection volume of 20 μl, standard curve method was used to calculate drug concentration in release media and the cumulative release profiles were obtained afterward.

The similarity factor (*f_2_*) can be used as an evaluation parameter of dissolution difference between the RLD and the generic preparation. The *f_2_* calculation formula is:

$$f_{2}=50\times log\left\{ \left[ 1+\frac{1}{n}\sum_{t=1}^{n} \left( R_{t}-T_{t} \right)^{2} \right]^{-\frac{1}{2}}\times100 \right\}$$

Where n represents the number of dissolution time points considered and *R_t_* and *T_t_* are the percent drug dissolved for the reference and test formulations at time point *t*. The value of *f_2_* ranges from 0 to 100 and dissolution profiles are considered similar when the *f_2_* value exceeds 50.

*Dose dumping induced by ethanol in vitro method:*

The drug release behaviors were investigated to 12 dosage units of DM SRT in a media of hydrochloric acid solution containing 5%, 20% and 40% ethanol or without ethanol at the conditions of *in vitro* dissolution test. Aliquots of 5 ml were sampled at 0.25, 0.5, 0.75, 1, 1.25, 1.5, 1.75, 2, 4, 6 and 8 h, respectively, and equal volume of fresh dissolution media at the same temperature was immediately added. The samples were filtered by 0.45 μm microporous membrane and determined according to chromatographic conditions depicted in “2.2 *in vitro* dissolution test”. The cumulative release rate was determined using the standard curve and the dissolution profiles were plotted.
